# A polynomial time algorithm for computing the area under a GDT curve

**DOI:** 10.1186/s13015-015-0058-0

**Published:** 2015-10-26

**Authors:** Aleksandar Poleksic

**Affiliations:** Department of Computer Science, University of Northern Iowa, 305 ITTC, Cedar Falls, Iowa, 50613 USA

**Keywords:** Protein structure, Structure modeling, Structure prediction, Model quality

## Abstract

**Background:**

Progress in the field of protein three-dimensional structure prediction depends on the development of new and improved algorithms for measuring the quality of protein models. Perhaps the best descriptor of the quality of a protein model is the *GDT* function that maps each distance cutoff *θ* to the number of atoms in the protein model that can be fit under the distance *θ* from the corresponding atoms in the experimentally determined structure. It has long been known that the area under the graph of this function (*GDT*_*A*) can serve as a reliable, single numerical measure of the model quality. Unfortunately, while the well-known *GDT*_*TS* metric provides a crude approximation of *GDT*_*A*, no algorithm currently exists that is capable of computing accurate estimates of *GDT*_*A*.

**Methods:**

We prove that *GDT_A* is well defined and that it can be approximated by the Riemann sums, using available methods for computing accurate (near-optimal) *GDT* function values.

**Results:**

In contrast to the *GDT_TS* metric, *GDT*_*A* is neither insensitive to large nor oversensitive to small changes in model’s coordinates. Moreover, the problem of computing *GDT*_*A* is tractable. More specifically, *GDT*_*A* can be computed in cubic asymptotic time in the size of the protein model.

**Conclusions:**

This paper presents the first algorithm capable of computing the near-optimal estimates of the area under the *GDT* function for a protein model. We believe that the techniques implemented in our algorithm will pave ways for the development of more practical and reliable procedures for estimating 3D model quality.

## Background

Advances in the area of protein three-dimensional structure prediction depend on the ability to accurately measure the quality of a protein model. One of the most popular and most reliable measure of the protein model quality is *GDT*_*TS*. It is defined as the average value of *GDT_P*_*θ*_ computed for four distance cutoffs $\theta = 2^{i}$, $i = \overline{0,3}$, where *GDT_P*_*θ*_ is the percentage of model residues (represented by their *C*_*α*_ atoms) that can be placed under *θ* ångströms from the corresponding residues in the experimental structure [1, 2]. In a “high-accuracy” version of *GDT*_*TS*, denoted by *GDT*_*HA*, the distance cutoffs are cut in half ($\theta = 2^{i}$, $i = \overline{ - 1,2}$) [3]. In both approaches, the underlying assumption is that the experimental (crystallographic or NMR) structure is close to the real (native) structure (which is sometimes not true due to experimental errors).

Several methods exist for computing *GDT*_*TS*. The LGA algorithm [4] can estimate *GDT*_*TS* quickly, but those estimates deviate from the true *GDT*_*TS* values in about 10 % of the cases [5]. Rigorous algorithms for computing *GDT*_*TS* have also been developed [6–9], but they are computationally much more expensive.

The *GDT*_*TS* is commonly interpreted as an approximation of the area under the *GDT* curve, denoted by *GDT*_*A* [10–12]. Unfortunately, since the measure is approximated using the *GDT* function values at only several distance cutoffs, the errors in the area approximation are large. As we demonstrate later, *GDT*_*TS* is not only overly sensitive to small but also insensitive to large changes in the protein model’s coordinates.

In this paper, we present a polynomial time algorithm for computing *GDT*_*A*. Our method runs on the order $\widetilde{O}(n^{3} )$, where *n* represents the length of the protein model (and $\widetilde{O}$ hides the log factor). The algorithm returns “near-optimal” *GDT*_*A* scores, meaning that the errors in our estimates can be made arbitrary small i.e., smaller than any upfront specified vale. Although our method is theoretical, we believe that its parallel implementations, coupled with carefully designed speed up techniques, can result in a practical and widely used software tool.

The rest of this paper is structured as follows. First, we present three examples that illustrate drawbacks of *GDT*_*TS* and advantages of *GDT*_*A*. Then, we place our theory on a firm mathematical ground, which enables us to formally define the *GDT*_*A* computation problem. Finally, we describe the actual algorithm for *GDT*_*A* and provide its running time analysis.

## Methods

### Definition of the GDT function

**Fig. 1:**
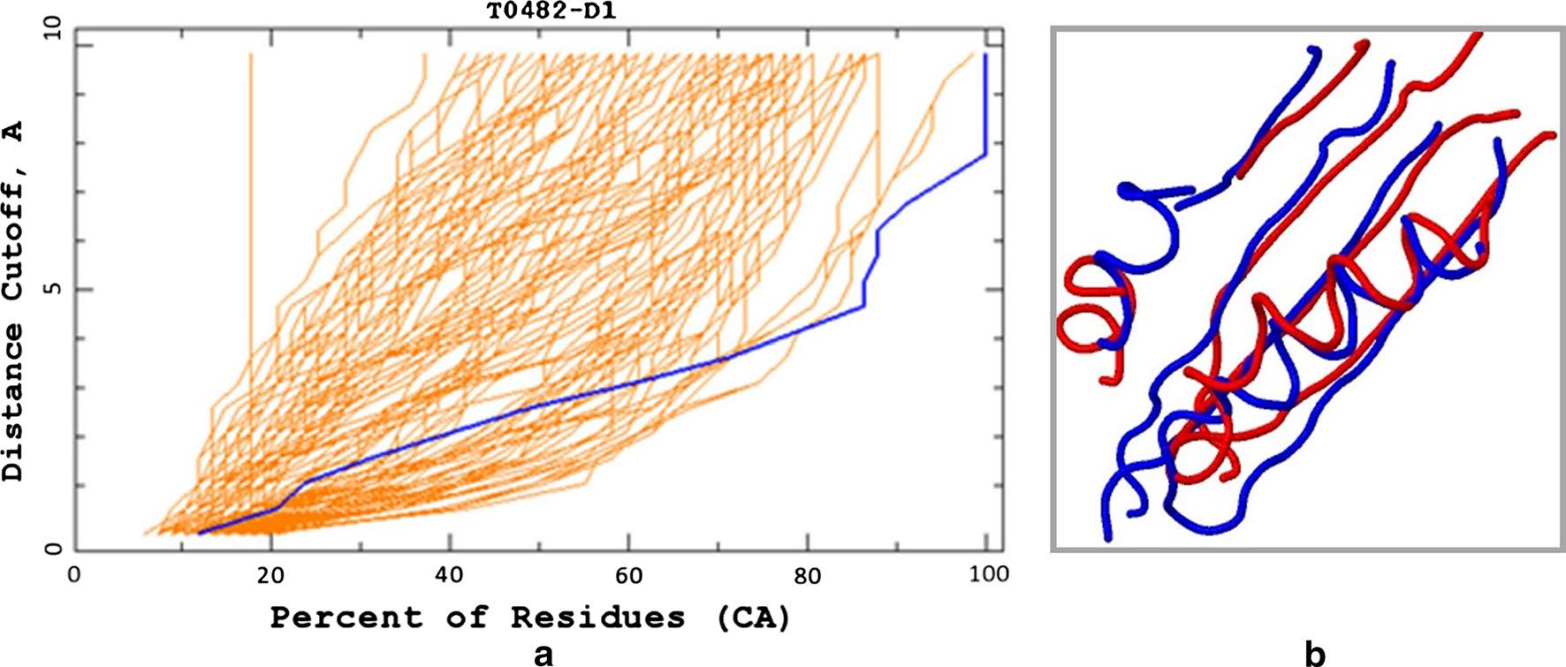
CASP 8 example. **a** *Red lines* represent *GDT* plots of different 3D models of the target protein T0482 submitted during the CASP8 experiment. The model submitted by the group TS208 is represented in *blue*. **b** Structural alignment of the model submitted by the group TS208 (*blue*) and the experimental structure (*red*) over 67 residues evaluated by the CASP assessors

The *GDT* function is a mapping that relates each distance cutoff *θ* to the percentage of model residues that can be placed at distance ≤*θ* from the corresponding residues in the experimentally determined structure. The graph of a *GDT* function provides a valuable insight into the quality of a protein model (Fig. 1). More specifically, the closer the graph runs to the horizontal axis (in other words, the smaller the area under the graph), the better the model.

As a single numerical measure of the model quality, *GDT*_*TS* is extensively used at CASP to rank different models for the same target [13, 14]. Since it represents the average of *GDT*_*P*_*θ*_ at several distance cutoffs, *GDT*_*TS* is often viewed as an approximation of the area under the *GDT* curve (*GDT*_*A*) [10–12]:

1 $$GDT\_TS = \sum\limits_{i = 0}^{3} {GDT\_P_{{2^{i} }} } .$$

However, as we demonstrate below, such a sparse sampling of the values of *GDT* function compromises the reliability of *GDT*_*TS*.

In our first example, we analyze the protein model for the target T0482, submitted by the group TS208 at CASP8 (Fig. 1). The *GDT*_*TS* score of this particular model was not even among the best dozen at CASP8, despite the fact that it fits the largest number of residues at distance $\le\sim 4$ from the corresponding residues in the experimental structure. In fact, the blue model (Fig. 1a) can be superimposed onto the experimental structure so that all of its residues are at distance $\le 8$ from the residues in the experimental structure (Fig. 1b), while no such superposition exists for any other model, even for the distance cutoff of 10Å. Interestingly, according to the MAMMOTH algorithm [15], the blue model is the best model for this particular target, while the DALI [16] algorithm ranks it as the second best.

Although it is impossible to tell whether #13 *GDT*_*TS* rank is more or less fair than #1 and #2 rank assigned by MAMMOTH and DALI, respectively, it is also not difficult to see that the ranking by the area under the *GDT* plot (*GDT*_*A*) would serve as a good compromise between these extremes.

**Fig. 2:**
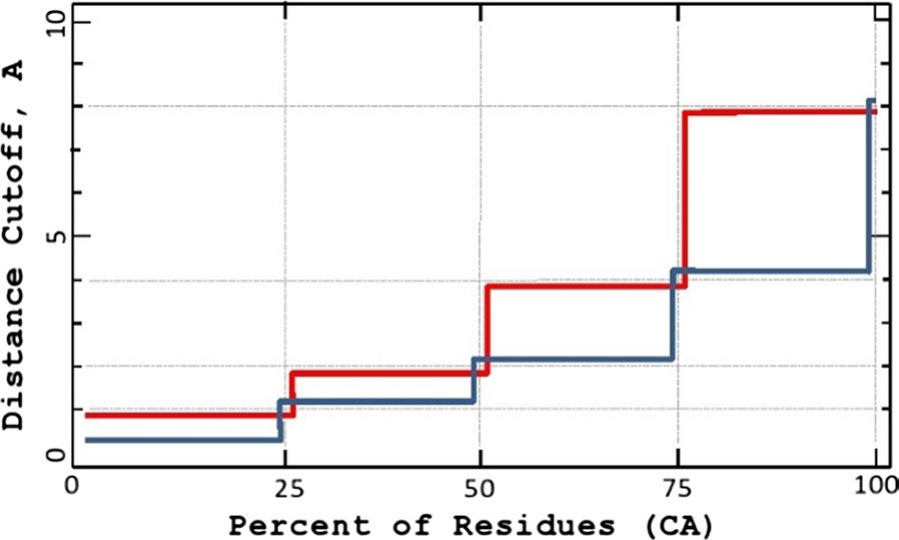
Insensitivity of GDT_TS. This theoretical example shows no sensitivity of *GDT*_*TS* to large variations in model quality. Surprisingly, the *red model* has a better *GDT*_*TS* score than the better *blue model*, even though it is worse by all standards. Notice that, unlike *GDT*_*TS*, the *GDT*_*A* measure is not skewed by the values at the cutoff points 1, 2, 4 and 8 Å. In fact, the *GDT*_*A* score of the blue model is twice as good as that of the *red model*

**Fig. 3:**
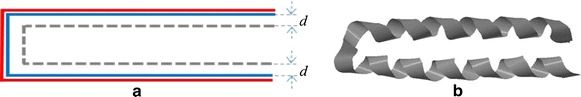
Oversensitivity of GDT_TS. **a** A four helix bundle-like (toy) protein (*dashed grey line*) along with two of its, almost identical, models (*red* and *blue*). A realistic example of such a target protein (PDB ID: 1JM0A) is shown on the *right* (**b**). In this example, we assume that the protein and its models are extended to the right to include 100 or more residues. Note that, if *d* ∈ {1 Å, 2 Å, 4 Å, 8 Å} then the *GDT* score of the *blue model* is significantly higher than that of the *red model*. For instance, if *d* = 2 Å, then the blue model has the *GDT_TS* score of about 87.5 since **~**50 % all of its residues can be fit at distance ≤1 Å and 100 % under each distance 2, 4 and 8 Å from the corresponding residues in the experimental structure (*dashed grey*). On the other hand, the *GDT_TS* score of the *red model* is only about 75, since only ~50 % of the *red model’s* residues can be placed under 1 and 2 Å and 100 % under 4 and 8 Å. In fact, no matter how close the *red model* gets to the *blue model*, its *GDT_TS* score will never improve. Note also that the *blue* and *red* *models* have almost identical *GDT_A* scores, since *GDT_A* is not sensitive to small coordinate changes

The next two examples illustrate further disadvantages of *GDT*_*TS*. As seen in Fig. 2, better *GDT*_*TS* scores can be assigned to obviously worse models. Moreover, as demonstrated in Fig. 3, very similar models can have significantly different *GDT*_*TS* scores.

### Mathematical formalism

**Fig. 4:**
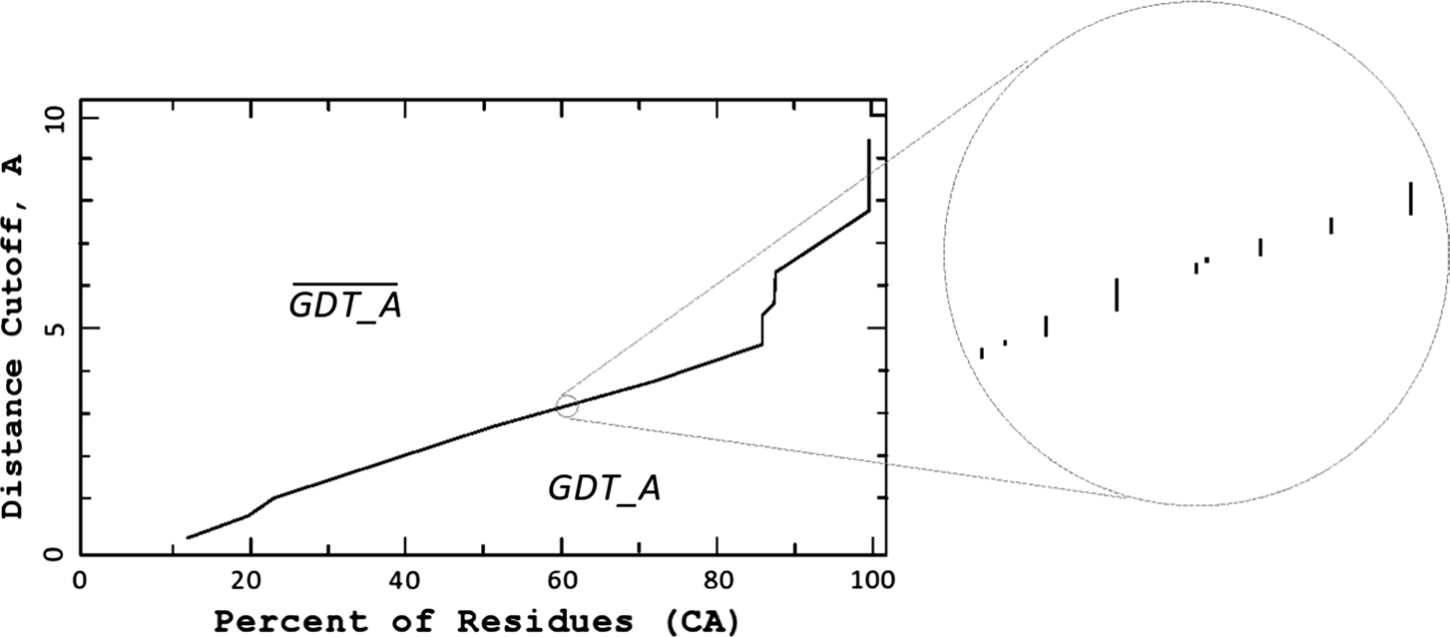
A closer look at the GDT_TS function. Zooming into the *GDT* plot of the model highlighted in Fig. 1. What appears to be the graph of a continuous function is, in fact, a set consisting of many separated *vertical line* segments

Strictly speaking, the *GDT* function is not well-defined. Zooming into the plot of the model highlighted in Fig. 1a, we see a set of many small vertical segments, meaning that each point on the horizontal axis is mapped to zero or more points on the vertical axis (Fig. 4). On the other hand, the inverse function (mapping each distance cutoffs *θ* to the percentage of residues in the model structure that can be fit under the distance *θ* from the corresponding residues in the experimental structure) is obviously well defined. This allows us to define the area under the *GDT* plot as the complement of the area under the inverse function:

2 $$GDT\_A = Total\_Area - \overline{GDT\_A}$$

where *Total*_*Area* represents the area of the rectangular region under consideration (100 × 10). We start our mathematical formalism by first defining a protein structure.

#### **Definition 1**

A *protein structurea* is a sequence of points in the three dimensional Euclidean space ${\mathbb{R}}^{3}$

3 $$a = (a_{1} , \ldots , a_{n} ).$$

The sequence elements *a*_*i*_ can represent individual atoms, but it is more typical (in particular in protein structure prediction experiments) to assume that each point *a*_*i*_ corresponds to the alpha-carbon atom of the protein’s *ith* amino acid.

In what follows, we formally define the *GDT* function [17]. For simplicity of presentation, we will modify the codomain of *GDT* to represent the “fraction of residues” (ranging from 0 to 1) instead of “percentages of residues” (ranging from 0 to 100). We note that this simple rescaling of the ordinate values will have no effects on the results obtained in our study.

#### **Definition 2**

Let $a = (a_{1} , \ldots , a_{n} )$ be a protein structure consisting of *n* amino acids, let $b = (b_{1} , \ldots , b_{n} )$ be a 3D model of *a*, and let $\overline{\theta }$ be a positive constant. The *Hubbard function (or GDT function)* is the function $H_{b} :[0,\overline{\theta } ] \to (0,1]$, defined by $H_{b} \left( \theta \right) = \mathop {\hbox{max} }\nolimits_{\tau } | \{ i \text{ }|\text{ } \| a_{i} - \tau \left( {b_{i} } \right) \|\le \theta \} |/n$, where  denotes the Euclidean norm on ${\mathbb{R}}^{3}$ and *τ* is a rigid transformation (a composition of a rotation and a translation).

#### **Theorem 1**

*H*_*b*_*is a stepwise function with finitely many steps*$\theta_{1} , \ldots , \theta_{k}$, 1 ≤ *k* ≤ *n* − 1.

#### *Proof*

Since *H*_*b*_ is monotony non-decreasing and since the range of *H*_*b*_ is a finite subset of (0,1], it follows that *H*_*b*_ must be a stepwise function. To complete the proof, we note that the number of steps in *H*_*b*_ matches the size of its range, which does not exceed *n* − 1, where *n* is the length of *b*.

For simplicity of presentation, from now on (and whenever the model *b* is implied), we will omit the subscript in *H*_*b*_ and denote the Hubbard function only by *H*.

### Algorithm for GDT_A

**Fig. 5:**
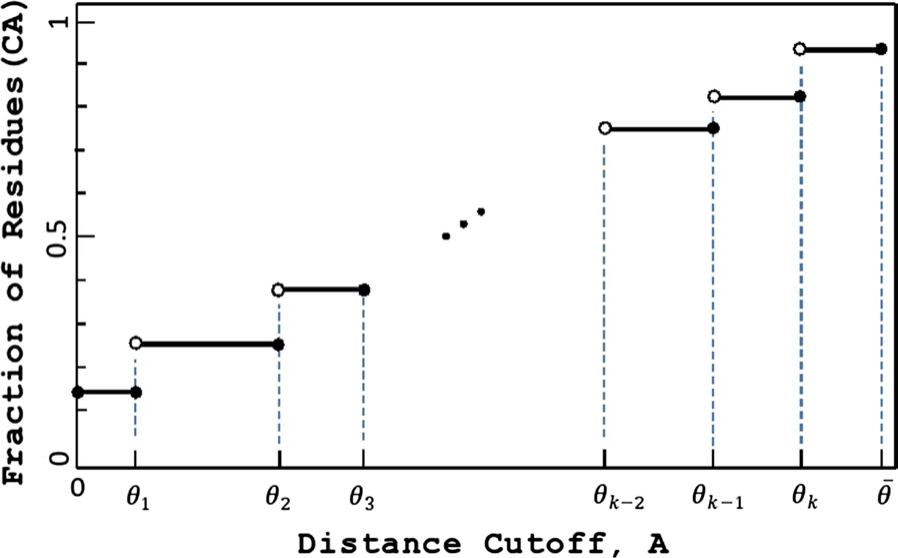
The general shape of the Hubbard function. Notice that the values *θ*_*i*_ along with the function values *H*_*b*_(*θ*_*i*_), $i = \overline{1,k}$, uniquely determine the area under the graph of *H*_*b*_. At the biannual CASP experiment, $\bar{\theta } = 10$

The area under *H* is the sum of the areas of the rectangular regions (*θ*_*i*_)(*θ*_*i*_ − *θ*_*i*−1_):

4 $$Area = \sum\limits_{i = 1}^{k + 1} {H(\theta_{i} )(\theta_{i} - \theta_{i - 1} )} ,$$

where *θ*_0_ = 0 and $\theta_{k + 1} = \overline{\theta }$ (Fig. 5). It would be trivial to compute *Area* had we known all *θ*_*i*_ and all function values *H*(*θ*_*i*_). Unfortunately, even if we knew the step points *θ*_*i*_, it would be computationally very difficult to compute the function values at them, since the best to date algorithm for computing *H*(*θ*_*i*_) runs on the order of *O*(*n*^7^) [7]. Hence, we resort to using the Riemann sums to approximate (instead of to compute exactly) the area under the graph of *H*.

The following definition and an accompanying theorem can be found in virtually any mathematical analysis textbook.

#### **Definition 3**

If $f:\left[ {a,b} \right] \to {\mathbb{R}}$ is a function then $R = \sum\limits_{i = 1}^{n} {v_{i} \left( {x_{i} - x_{i - 1} } \right)},$ where *a* = *x*_0_ < *x*_1_ < ··· < *x*_*n*_ = *b* is the partition of the interval [*a*, *b*] and *v*_*i*_ denotes the supremum of *f* over [*x*_*i*−1_, *x*_*i*_], is called the *upper Riemannsum* of *f* on [*a*, *b*].

#### **Theorem 2**

*Let f be a real, non-decreasing, Riemann integrable function on an interval* [*a*, *b*]. *Then*

5 $$\left| {\int_{a}^{b} {f\left( x \right)dx - R} } \right| < \Delta x\left( {f\left( b \right) - f\left( a \right)} \right),$$

*where*

6 $$R = \sum\limits_{i = 1}^{n} {v_{i} \left( {x_{i} - x_{i - 1} } \right)}$$

*is the upper Riemann sum of f the and*$\Delta x = \max_{i} (x_{i} - x_{i - 1} ).$

Observe that, since *H*_*b*_ is piecewise continuous, it must be integrable on $[0,\overline{\theta } ]$. Thus, the area under the graph of *H* is

7 $$Area = \mathop \int \nolimits_{0}^{{\overline{\theta } }} H\left( \theta \right)d\theta .$$

**Fig. 6:**
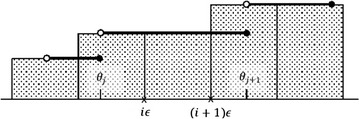
Approximation of the Hubbard function by the Riemann sum. *Area*($\epsilon$) is the sum of the areas of all rectangular regions

To approximate *Area* with a Riemann sum, one can define the partition points $\epsilon ,2\epsilon , \ldots ,m\epsilon$, where $m = \lceil {\overline{\theta}} / \epsilon \rceil$ (Fig. 6) and then compute an estimate *Area*($\epsilon$) of *Area* as

8 $$Area (\epsilon)= \sum_{i=1}^m \epsilon H(i\epsilon)$$

The error |*Area* – *Area* ($\epsilon$)| in the estimate (8) is below 2$\epsilon$. Up to a half of this error is due to the error in the Riemann sum with the remaining error being due to the possible placement of the last partition point *m*$\epsilon$ outside the interval $[0,\overline{\theta } ]$.

Unfortunately, computing the area estimates according to (8) is still a challenging problem, because (as we mentioned above), there is no computationally effective procedure for finding the function values *H*(*i*$\epsilon$). To circumvent the problem, we utilize an efficient algorithm capable of computing the lower bound estimates *H*_*i*_ of *H*(*i*$\epsilon$), satisfying $H\left( {(i - 1)\epsilon} \right) \le H_{i} \le H\left( {i\epsilon} \right)$, $i = \overline{1,m}$. We then compute an estimate $\widetilde{Area}(\epsilon)$ of *Area* as

9 $$\widetilde{Area}\left( \epsilon \right) = \mathop \sum \limits_{i = 1}^{m} \epsilon H_{i}.$$

Since $\left| {\widetilde{Area}\left(\epsilon\right) - Area(\epsilon)} \right| < 2\epsilon$, it follows that $\widetilde{Area}(\epsilon)$ is a $4 \epsilon$-approximation of *Area*. Below we show how to compute all *H*_*i*_’*s*, and, in turn, $\widetilde{Area}(\epsilon)$ in time $O\left({n^{3} logn/\epsilon^{6} } \right)$, where *n* is the length of *b*. Our algorithm takes advantage of an efficient procedure for computing near optimal *GDT*_*TS* values [5].

Let *T*(*b*) denotes the image of the model structure *b* under the transformation *T*. Denote by $MAX(T, \theta )$ the largest fraction of residues from *T*(*b*) that are at distance ≤*θ* from the corresponding residues in the experimental structure *a*. To find each *H*_*i*_, it is enough to compute a rigid body transformation *T*_*i*_ satisfying $H\left({(i - 1)\epsilon} \right) \le MAX\left({T_{i} ,i}\epsilon \right) \le H\left({i} \epsilon\right)$.

Denote by *T*_*θ*_ a transformation that places a largest subset *b*_*θ*_ of residues from *b* at distance ≤*θ* from the corresponding residues in the experimental structure. Given *T*_*θ*_, one can easily compute *b*_*θ*_ by calculating all *n* distances between the residues *a*_*i*_ and *T*_*θ*_(*b*_*i*_). Note that *P*(*T*_*θ*_, *θ*) = *H*(*θ*). We approximate the transformation *T*_*θ*_ by a so-called “near-optimal” transformation i.e., a transformation that places at least as many residues from the model structure under distance $\theta + \epsilon$ as the optimal transformation *T*_*θ*_ places under the distance *θ*. From now on, we will use $T_{\theta}^{\epsilon}$ to denote a “near-optimal” transformation and the corresponding set of residues will be denoted by $b_{\theta}^{\epsilon}$. Observe that $P\left({T_{\theta}^{\epsilon}, \theta + \epsilon} \right) \ge P\left({T_{\theta}, \theta} \right) = H(\theta)$.

Building upon any procedure for computing $T_{\theta}^{\epsilon}$, one can develop an algorithm for $\widetilde{Area}(\epsilon)$ by substituting $P\left({T_{{\theta_{i}}}^{\epsilon}, \theta_{i} + }\epsilon \right)$ for *H*_*i*_ in (10), where $\theta_{i} = (i - 1)\epsilon$. Several existing methods can be modified and made suitable for finding $T_{\theta}^{\epsilon}$. The most efficient such method relies on the concept of “radial pair” [5].

#### **Definition 4**

Let $S = \left\{ {s_{1} , \ldots , s_{n} } \right\}$ be a set of points in the three-dimensional Euclidean space. An ordered pair of points $(s_{i} , s_{j} )$ is called a *radial pair* of *S* if *s*_*j*_ is the furthest point from *s*_*i*_ among all points in *S*.

#### **Theorem 3**

*Let T*_1_* andT*_2_*be two transformations and let*$(s_{k} , s_{l} )$* be a radial pair of S*. *If*$\|T_{1} \left({s_{k}} \right) - T_{2} (s_{k})\| <\epsilon /3$*and*$\|T_{1} \left({s_{l}} \right) - T_{2} (s_{l})\| < \epsilon/3$* then there exists a rotation Raround the line through T*_1_(*s*_*k*_) *and*$T_{1} (s_{l} )$*such that*$\|R\left({T_{1} \left({s_{p} } \right)} \right) - T_{2} (s_{p}) \|<\epsilon$, *for any s*_*p*_* inS*. *The rotation R can be found in time O* (*nlogn*), *where n is the size of S*.

A proof of the above theorem can be found in [5]. The algorithm for finding *R* is fairly straightforward and it relies on the so-called *plane*-*sweep* approach [18].

The Theorem 3 implies that one choice for the near-optimal transformation $T_{\theta}^{\epsilon}$ is the transformation *R* ∘ *T*, where *T* is any transformation that maps the points *b*_*k*_ and *b*_*l*_ from the radial pair (*b*_*k*_, *b*_*l*_) of *b*_*θ*_ to the $\epsilon/3$ neighborhoods of *T*_*θ*_(*b*_*k*_) and *T*_*θ*_(*b*_*l*_), respectively, and *R* is the rotation around the radial axis $\overline{{T\left( {b_{k} } \right)T\left( {b_{l} } \right)}}$ that maps the remaining points from *T*(*b*_*θ*_) to the $\epsilon$-neighborhoods of the corresponding points from *T*_*θ*_(*b*_*θ*_).

In search for a radial pair of *b*_*θ*_, the algorithm in [5] explores all *n*^2^ possible pairs of residues in *b*. For each candidate radial pair $(b_{k} , b_{l} )$, the algorithm generates a finite, representative set of transformations that map *b*_*k*_ and *b*_*l*_ into $\theta + \epsilon/3$ neighborhoods of *a*_*k*_ and *a*_*l*_, respectively (see the paragraph below for more details). For every such transformation *T*, a plane-sweep algorithm [18] is used to find a rotation *R* around the axis $\overline{{T\left( {b_{k} } \right)T\left( {b_{l} } \right)}}$ that maximizes the number of residues from *R*(*T*(*b*)) that can be placed at distance $<\theta + \epsilon$ from the corresponding residues in *a*.

**Fig. 7:**
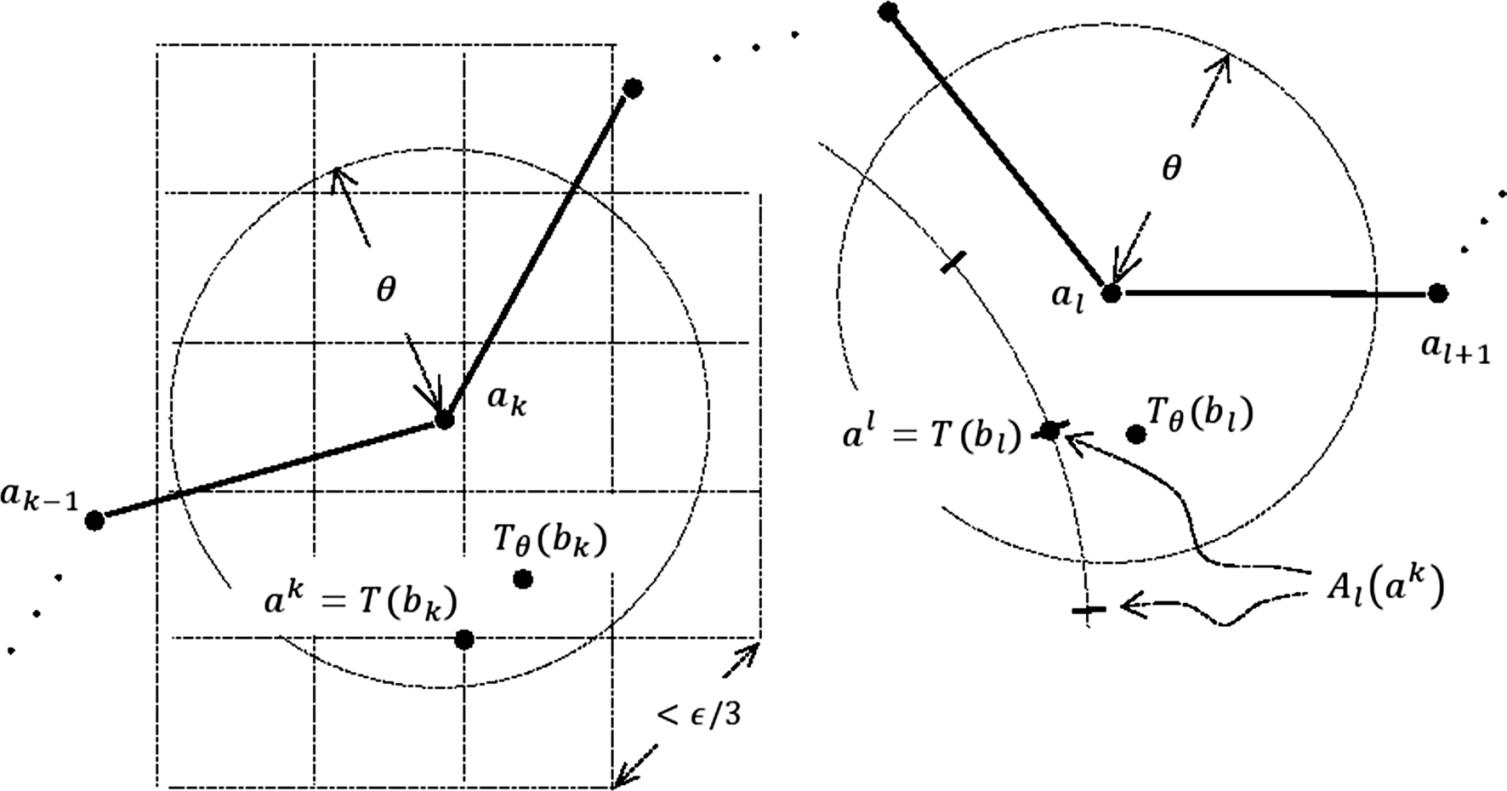
Discretizing the space of rigid body transformations. 2D illustration of *A*_*k*_ (the set of the vertices of the *squares* shown on the *left*) and the set *A*_*l*_ (*a* ^*k*^) generated for one *a*_*k*_ ∈ *A*_*k*_

A finite set of transformations that map the residues *b*_*k*_ and *b*_*l*_ into the $\theta + \epsilon/3$ neighborhoods of *a*_*k*_ and *a*_*l*_, respectively, is constructed in such a way to ensure that for at least one of those transformation *T*, $\| T\left({b_{k}} \right) - T_{\theta} (b_{k} )\| < \epsilon/3$ and $\|T\left({b_{l}} \right) - T_{\theta} (b_{l}) \|< \epsilon/3$. This can be achieved by partitioning ${\mathbb{R}}^{3}$ into small cubes of side length slightly smaller than $\sqrt 3\epsilon /9$ and then collecting the vertices of the cubes that are inside the open ball of radius $\theta + \epsilon/6$ around *a*_*k*_ (Fig. 7). The elements of this set, denoted by *A*_*k*_, are the candidate points *T*(*b*_*k*_). The number of points in *A*_*k*_ is $O(1/\epsilon^{3} )$ and at least one of them must be at distance $<\epsilon/6$ from *T*_*θ*_(*b*_*k*_) (Fig. 7). For each point *a*^*k*^ ∊ *A*_*k*_, the set *A*_*l*_(*a*^*k*^) of possible images of *b*_*l*_ under *T* is computed by discretizing the spherical cap $S(a^{k}, \| b_{k} - b_{l}\| ) \cap B(a_{l} ,\theta + \epsilon/3)$, where *S*(*a*, *r*) and *B*(*a*, *r*) denote the sphere and the open ball in ${\mathbb{R}}^{3}$ with center *a* and radius *r*, respectively, in such a way that at least one point from *A*_*l*_(*a*^*k*^) is found at distance $< \epsilon/3$ from *T*_*θ*_(*b*_*l*_) (Fig. 7). We note that size of *A*_*l*_(*a*^*k*^) is $O(1/\epsilon^{2} )$. Hence, the total number of candidate pairs of points (*T*(*b*_*k*_), *T*(*b*_*l*_)) is $O(1/\epsilon^{5})$.

An obvious to compute $T_{{\theta_{1} }}^{\epsilon} , \ldots ,T_{{\theta_{m} }}^{\epsilon}$ is to run the just described algorithm *m* times in succession, for $\theta = \theta_{1} , \ldots , \theta = \theta_{m}$. However, such an approach results in many unnecessary repeated calculations as the area around *a*_*k*_ and the corresponding spherical cap in the neighborhoods of *a*_*l*_ are discretized over and over again. Moreover, all transformations *T* and *R*, generated and inspected during the procedure for finding $T_{{\theta_{i}}}^{\epsilon}$, are inspected again during the procedure for finding $T_{{\theta_{j}}}^{\epsilon}$, for each *j* > *i*. 

We show that all transformations $T_{{\theta_{1}}}^{\epsilon} , \ldots ,T_{{\theta_{m} }}^{\epsilon}$ and the corresponding values *H*_1_, …, *H*_*m*_ can be computed, at once, during the procedure of finding the last transformation, namely $T_{{\theta_{m} }}^{\epsilon}$. As demonstrated in the pseudocode above, the transformation *T* is generated only once for each pair of points $\left( {a^{k} , a^{l} } \right) \in A_{k} \times A_{l} \left( {a^{k} } \right)$ and a sweep-plane algorithm for finding *R* is called only once for each *i* satisfying $\|a_{k} - a^{k}\| < \theta_{i} + \epsilon/6$ and $\|a_{l} - a^{l} \|< \theta_{i} + \epsilon/3$. The values of *H*_*i*_ are updated on the fly.

### Running time analysis

To analyze the algorithm’s running time, we note that the number of iterations of the first* for* loop is equal to the number of candidate radial pairs (*b*_*k*_, *b*_*l*_), which is $O\left( {n^{2} } \right).$ The number of iterations of the second *for* loop matches the number of pairs of grid points around *a*^*k*^ and *a*^*l*^, which is $O\left( {1/\epsilon^{3} } \right) \times O\left( {1/\epsilon^{2} } \right) = O\left( {1/\epsilon^{5} } \right)$. Each one of $O\left( m \right) = O(\lceil\bar{\theta }/\epsilon\rceil) = O(1/\epsilon)$ iterations of the third *for* loop calls a *O*(*nlogn*) plane-sweep procedure to compute an optimal rotation and (if needed) to update the value *H*_*i*_. Hence, the asymptotic time complexity of the three nested *for* loops is $O\left( {n^{3} logn/\epsilon^{6} } \right).$

## Conclusions

Estimating the quality of a protein 3D model is a challenging task. Automatically generated *GDT*_*TS* score is helpful as the first raw approximation but this measure is neither sensitive nor selective enough to be exclusively relied upon in ranking different models for the same target. In this paper, we show that using a more accurate approximation of the area under the *GDT* curve as the criterion of model quality addresses many of the drawbacks of *GDT*_*TS*. We also present a rigorous $\widetilde{O}\left( {n^{3} } \right)$ algorithm for computing the area under the *GDT* curve for a given model, where *n* is the model’s length. The area estimate returned by our method is “near-optimal”, meaning that the error in the estimate can be made smaller than any upfront specified value.

Despite the cubic asymptotic running time with a relatively large hidden constant, we believe that the techniques presented in this paper can guide a future development of a computationally efficient computer program, in particular since our methodology is amenable to parallel implementations. A heuristic version of the algorithm for estimating the area under the *GDT* plot can be found at http://bioinfo.cs.uni.edu/GDT_A.html.
